# Understanding the phenotypic impact of non-coding sequence variation

**DOI:** 10.1186/1471-2164-15-S2-O12

**Published:** 2014-04-02

**Authors:** Emmanouil Dermitzakis

**Affiliations:** 1 Department of Genetic Medicine and Development, Suisse University of Geneva Medical School, 1 Rue Michel-Servet (CMU office 9088), Geneva 1211, Switzerland; 2 Center of Excellence in Genomic Medicine Research, King Abdulaziz University, P.O. Box: 80216 Jeddah 21589, Kingdom of Saudi Arabia

## 

Molecular phenotypes are important phenotypes that informs about genetic and environmental effects on cellular state. The elucidation of the genetics of gene expression and other cellular phenotypes are highly informative of the impact of genetic variants in the cell and the subsequent consequences in the organism. In this talk, I will discuss recent advances in three key areas of the analysis of the genomics of gene expression and cellular phenotypes in human populations and multiple tissues and how this assists in the interpretation of regulatory networks and human disease variants. I will also discuss how the recent advances in next generation sequencing and functional genomics are bringing closer our hopes for personalized medicine.

